# Enabling Genomic-Phenomic Association Discovery without Sacrificing Anonymity

**DOI:** 10.1371/journal.pone.0053875

**Published:** 2013-02-06

**Authors:** Raymond D. Heatherly, Grigorios Loukides, Joshua C. Denny, Jonathan L. Haines, Dan M. Roden, Bradley A. Malin

**Affiliations:** 1 Department of Biomedical Informatics, School of Medicine, Vanderbilt University, Nashville, Tennessee, United States of America; 2 School of Computer Science and Informatics, Cardiff University, Cardiff, Wales, United Kingdom; 3 Department of Biomedical Informatics/Department of Medicine, School of Medicine, Vanderbilt University, Nashville, Tennessee, United States of America; 4 Department of Molecular Physiology and Biophysics/Center for Human Genetics Research, School of Medicine, Vanderbilt University, Nashville, Tennessee, United States of America; 5 Department of Medicine/Department of Pharmacology, School of Medicine, Vanderbilt University, Nashville, Tennessee, United States of America; 6 Department of Biomedical Informatics, School of Medicine/Department of Electrical Engineering and Computer Science, School of Engineering, Vanderbilt University, Nashville, Tennessee, United States of America; Tel Aviv University, Israel

## Abstract

Health information technologies facilitate the collection of massive quantities of patient-level data. A growing body of research demonstrates that such information can support novel, large-scale biomedical investigations at a fraction of the cost of traditional prospective studies. While healthcare organizations are being encouraged to share these data in a de-identified form, there is hesitation over concerns that it will allow corresponding patients to be re-identified. Currently proposed technologies to anonymize clinical data may make unrealistic assumptions with respect to the capabilities of a recipient to ascertain a patients identity. We show that more pragmatic assumptions enable the design of anonymization algorithms that permit the dissemination of detailed clinical profiles with provable guarantees of protection. We demonstrate this strategy with a dataset of over one million medical records and show that 192 genotype-phenotype associations can be discovered with fidelity equivalent to non-anonymized clinical data.

## Introduction

Routine clinical care generates detailed, longitudinal information about a patient’s health, medications, allergies, and treatment response. Recording and preserving these data, typically through an electronic medical record (EMR), can enable greater efficiency and effectiveness in the actions of care providers [1]–[3]. In the hopes of realizing the full potential of health information technology, the past several years has witnessed dramatic growth in the quantity and quality of clinical data [4], which, in turn, has become an invaluable resource for a wide range of secondary (i.e., not direct care) endeavors [5], [6], including public health [7], [8], quality assessment [9], and medical research [10], [11]. With regard to the latter, EMRs are increasingly linked to biorepositories to enable large cost-effective association studies between genomes and an expanding range of phenotypes [12]–[15], such as atrioventricular conduction [16], white [17] and red [18] blood cell traits, hypothyroidism [19], and, more recently, the study of pharmacogenetic traits, including clopidogrel-response [20] and warfarin dose [21]. To facilitate transparency and enable reuse, collections of genotypes and DNA sequences tied to clinical knowledge are shared beyond the originating healthcare institutions, such as through the Database of Genotypes and Phenotypes (dbGaP) at the National Institutes of Health [22].

The majority of datasets currently shared via dbGaP, and similar environments, enable validation of known findings [23], but they lack the phenotypic detail necessary to support novel scientific investigations, thus slowing or preventing innovative biomedical research. A major obstacle to dissemination of clinically-rich datasets is the concern that disclosure of detailed records can cause privacy breaches, particularly in the form of patient re-identification [24], [25]. Indeed, a growing number of studies illustrate how simple patient-specific data, such as demographics [26]–[29], hospital visit patterns [30], or insurance billing codes [31] – which correspond to International Classification of Diseases - 9th Revision (ICD-9) and are a core element of clinical phenotype specifications [13] – can be exploited for identification purposes. An additional concern is the contention that DNA sequence information is inherently identifiable [32], although patient-specific sequence databases to create such vulnerabilities are not (yet) generally available [33].

Concerns over re-identification can be mitigated through pragmatic governance models that integrate ethical, legal, and technical controls [34]–[37]. From a technical perspective, various approaches for the anonymization of patient-specific data have been proposed [38], [39], but they are limited in their scope by considering unrealistically strong attackers. Of particular importance for the dissemination of clinical data, Loukides et al. introduced an anonymization method for billing codes [40], but assumed the recipient of the data knows that a specific patient is a member of the cohort. While such a threat is plausible, it is not always likely and, in many situations, it is prudent for healthcare institutions to assume more realistic adversaries: for example, a recipient may only know that an individual was a patient at the hospital and not that they were a member of a specific research cohort [41], [42]. We hypothesize that using larger populations for anonymization will yield more accurate biomedical knowledge discovery.

To investigate this hypothesis, we developed methods to anonymize datasets that contain a large amount of clinical data that account for varying degrees of a recipient’s knowledge. To assess our models, we conducted an evaluation with three datasets derived from the EMR system of the Vanderbilt University Medical Center (VUMC), covering over one million patient records. Our findings illustrate that making more pragmatic assumptions on the capabilities of the recipient enables the dissemination of significantly greater quantities of patient-specific data in comparison to prior approaches. We find this method enables the dissemination of privacy-protected clinical data that support the discovery of phenome-wide associations equal to those previously published using non-protected information [43].

## Results

We evaluated the influence of anonymization on two distinct types of knowledge discovery criteria. First, we summarize the quantity of clinical information retained in the anonymized datasets in comparison to the original resource. This provides a general sense of the quantity of clinical knowledge that can be disseminated. Second, we conducted Phenome-wide Association Studies (PheWAS) to characterize the extent to which phenotype-genotype associations are retained. In this scenario, all of our assessments are performed on the DEMO dataset.

### Retention of General Clinical Information

Table 1 summarizes the quantity of clinical information retained in the anonymized datasets. We represent the changes through the use of two measures: Diagnosis Coverage (DC) and Code Coverage (CC). Diagnosis Count is a general measure of how many unique diagnoses are contained in the anonymized data, while Code Count is a measure of how many unique ICD-9 codes appear in the anonymized data. First, we examine the changes to the datasets resulting from anonymization process. It can be seen that *SD-Anon* yields the best retention of clinical information (99.99% DC and 99.98% CC) and *BioVU-Anon* has a slightly higher DC than 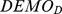 (99.99% and 99.57%, respectively). However, 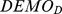 has a higher CC than *BioVU-Anon* (80.78% and 77.02%, respectively). It is worth noting that this finding is influenced by the difference in CC and DC in the initial subsets (i.e., *DEMO* and *BioVU*). If the counts are considered in relation to the original SD, then *BioVU-Anon* has a DC of 19.71% and a CC of 67.65%, while 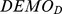 has a DC of 2.02% and a CC of 46.80%, showing that BioVU-Anon retains more information than 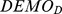 overall.

**Table 1:** Summary statistics and information retention for the datasets in this study.

Original Dataset*Anonymized Version*	Code Count		Diagnosis Count		Population Size
**Synthetic**					
Derivative(SD)*	**15,115**	**–**	**13,432,263**	**–**	**1,366,786**
*SD-Anon*	*15,112*	*99.98%*	*13,431,347*	*99.99%*	*1,366,552*
BioVU*	**13,275**	**–**	**2,647,056**	**–**	**104,904**
*BioVU-Anon*	*10,225*	*(77.02%)*	*2,646,872*	*(99.99%)*	*104,790*
**Demonstration**					
Group (DEMO)*	**8,734**	**–**	**272,080**	**–**	**5,994**
*DEMO_s_*	*8,747*	*(99.93%)*	*272,043*	*(99.99%)*	*5,994*
*DEMO_B_*	*8,476*	*(99.93%)*	*248,925*	*(91.50%)*	*5,595*
*DEMO_D_*	*7,071*	*(80.78%)*	*270,867*	*(99.57%)*	*5,971*

Code Count and Diagnosis Count are the number of unique ICD-9 (or generalized set of ICD-9 codes) and total number of diagnoses for all records in the anonymized dataset, respectively. In this table, *corresponds to the original (i.e., non-anonymized) datasets.

Next, we compare the information retained in the three Demonstration groups. Again, we see that 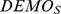, the SD anonymization, performs better than either of the other anonymizations (99.99% DC and 99.93% CC). Additionally, we find that 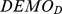 performs better than 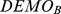 in DC (99.57% and 91.50%, respectively), whereas the reverse is true for CC (80.78% and 96.84%, respectively).

In combination, these findings partially confirm our earlier hypothesis. 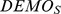, which is derived from the largest population, results in the best retention of general clinical information among the DEMO anonymizations. However, neither 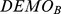 nor 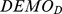 clearly outperforms the other, which we discuss below.

In Tables 2 and 3, we show the full results we measured following the anonymization. In Table 2, we report the expanded DC information. In the left part of the chart, we report our findings without generalization - that is, if codes which occur in fewer than *k* records are removed from the set, rather than aggregated. For this, we report three measures. First, the count of diagnoses in the data set. Second, the as a percentage of the count to the total number of diagnoses in the SD (SD%). This measure allows us to determine how much information this anonymization retains of the entire population data set. Finally, we report the ratio of the count to the total number of diagnoses in its similar, non-anonymized data set. For example, for *BioVU-Anon*, the Local % measure compares to *BioVU*. As we hypothesized, we see in that even without generalization, *SD-Anon* still represents the highest retention of data of the SD (99.97%). We further see that BioVU-Anon and 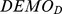 each have lower percentages of the SD, as is expected. However, we also see that they each contain less information with respect to their original dataset as well (99.71% and 99.20%, respectively).

**Table 2:** Full Diagnosis Count information retained.

Dataset	Diagnosis Count			Diagnosis Count		
	without Generalization			with Generalization		
	Count	SD %	Local %	Count	SD %	Local %
SD-Anon	13428542	99.97%	99.97%	13431347	99.99%	99.99%
BioVU-Anon	2639298	19.65%	99.71%	2643872	19.68%	99.88%
*DEMO_D_*	269868	2.01%	99.20%	270867	2.02%	99.57%
*DEMO_s_*	271970	2.02%	99.97%	272043	2.03%	99.99%
*DEMO_B_*	248467	1.85%	91.33%	248925	1.85%	91.50%

**Table 3:** Full Code Count information retained.

Dataset	Code Count			Code Count		
	without Generalization			with Generalization		
	Count	SD %	Local %	Count	SD %	Local %
SD-Anon	13525	89.48%	89.48%	15112	99.98%	99.98%
BioVU-Anon	9785	64.74%	73.71%	10225	67.65%	77.02%
DEMOD	6952	45.99%	79.42%	7071	46.78%	80.78%
DEMOS	8681	57.43%	99.18%	8747	57.87%	99.93%
DEMOB	8179	54.11%	93.44%	8476	56.08%	96.84%

If we turn our attention to a comparison of the three Demonstration datasets, we see that 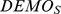 is still, as expected, the best performing. We note that our earlier observation about the divergence from our expectation degrading 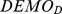 and 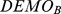 still holds true here.

Next, in Table 3, we show the extended CC information retained. Similarly to Table 3, we report the original count and use the same additional measures to determine the information retention.

Here, we note several interesting findings. First, while the DC without and with generalization (99.97%, 99.99%) were similar, this is not the case with CC. Without generalization, *SD-Anon* retains only 89.48% of codes, while with generalization this retention is 99.98%. Considering these two statistics together, we see that by generalizing, we are able to release information about 10.5% of codes - approximately 1,500 codes – that, while generalized in the release, would be completely absent from the data were we to simply suppress them. We see similar, though less dramatic, results from the other two data sets.

If we again turn our attention to a comparison of the three DEMO sets, we see that, again, 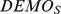 clearly has more information retention than the other two DEMO sets. Most strikingly, 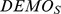, using the Local % measure, retains almost 30% more codes in the anonymized set (approximately 4,500 codes) over 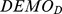 even without generalization. If we also consider generalized codes, 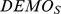 keeps approximately 19% more codes over 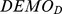.

Again, we note that the difference in retention between 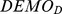 and 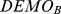 that was visible in the DC measure does not appear in the Code Count measure. Instead, we see that, as hypothesized, the larger the original set of data that is anonymized, the more information we retain in the anonymized data set (as measured by number of codes available for evaluation). Even though 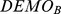 performs better using this measure, the clearly superior data set is 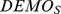.

### Retention of Genotype-Phenotype Associations

A Phenome-Wide Association Study (PheWAS) [19], [44], [45] assesses which clinical phenotypes from across a collection of concepts (in this case, a set of related billing codes grouped according to semantic similarity) are associated with a specific genomic region of interest. Patients are marked as either cases or controls according to the presence and absence of certain billing codes. In its simplest form the analysis determines the genotype distributions and calculates a 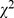 statistic, with an associated p-value and an odds-ratio. Conditions with 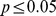 corrected for multiple comparisons are considered significant. We note that the point of the analysis in this paper is to determine the level of information loss resulting from anonymization of this dataset; specifically, we acknowledge that each of the conditions labeled as significant here only indicate a potential significance in a PheWAS discovery analysis, and are not necessarily conclusive. To conduct the present analysis, we focus on the anonymized demonstration cohorts and the six single nucleotide polymorphisms (SNPs) analyzed in the original PheWAS study of Denny et al. [43]. Table 4 compares the associations discovered in the anonymized and original datasets.

**Table 4:** Results of anonymization on PheWAS Analysis for six SNPs.

SNP	Phenotype Associations at 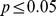 in PheWAS						
	Original number of associations	Lost Associations			False Associations		
		(Type I Error)			(Type II Error)		
		*DEMO_D_*	*DEMO_B_*	*DEMO_S_*	*DEMO_D_*	*DEMO_B_*	*DEMO_S_*
*rs1333049*	30	0	9	0	1	13	0
*rs2200733*	27	2	8	0	1	7	0
*rs2476601*	33	1	4	0	4	6	0
*rs3135388*	39	4	12	0	2	9	0
*rs6457620*	35	3	7	0	0	12	0
*rs17234657*	28	3	9	0	1	6	0
*Total*	192	13	49	0	9	53	0

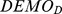
, 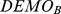, and 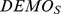 are the Demonstration group when anonymized, extracted from the BioVU anonymization, and extracted from the SD anonymization, respectively. Original is the number of significant associations (p 0.05) found in the PheWAS when conducted on pre-anonymized data. Identical is the number of associations which were the same between studies. Lost is the number of associations that were lost in the anonymized study. False is the number of associations that were determined as significant in the new study but were not in the original.

We first look at Type I errors, which we refer to as lost associations. These correspond to conditions determined to be significant in the original PheWAS study on DEMO, but were found to have 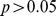 in an anonymized dataset. It can be seen that only 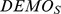 yields no lost associations across all SNPs. By contrast, 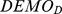 has only a single SNP (*rs1333049*) where there were no lost associations. Every other SNP has at least one lost association. 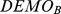 sustained lost associations in each SNP. Notably, 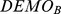 sustained a larger number of lost associations than 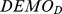 for every SNP.

Next, we turn our attention to Type II errors, which we refer to as false associations. These correspond to conditions with 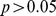 in the original study, but 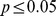 in the anonymized dataset. It can be observed that 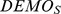 is the only anonymization which in all cases has no additional significant associations reported. Similarly, 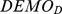 has one SNP (*rs6457620*) which has no new associations, though this is not the same SNP that sustained no lost associations. Again, 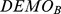 yielded new associations for each SNP.

### PheWAS Case Studies

**Figure 1:**
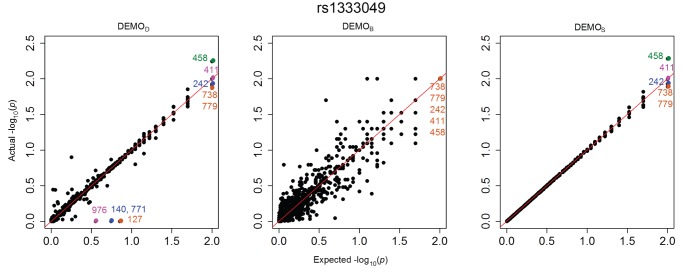
Changes in p-values for associations between clinical conditions and SNP rs1333049 presented as a QQ-plot for left) 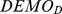, middle) 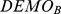, and right) DEMOS. Descriptions of the annotated conditions in the plots are provided in Table 5.

**Figure 2:**
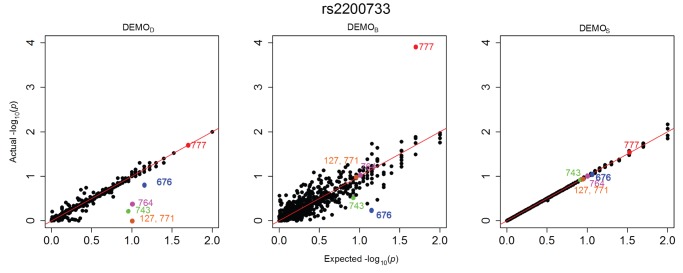
Changes in 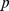-values for associations between clinical conditions and SNP rs2476601 presented as a QQ-plot for left) 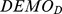, middle) 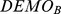, and right) DEMOS. Descriptions of the annotated conditions in the plots are provided in Table 5.

**Figure 3:**
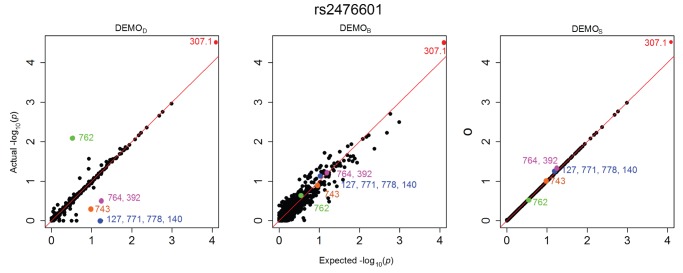
Changes in 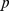-values for associations between clinical conditions and SNP rs2200733 presented as a QQ-plot for left) 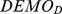, middle) 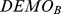, and right) DEMOS. Descriptions of the annotated conditions in the plots are provided in Table 5.

**Figure 4:**
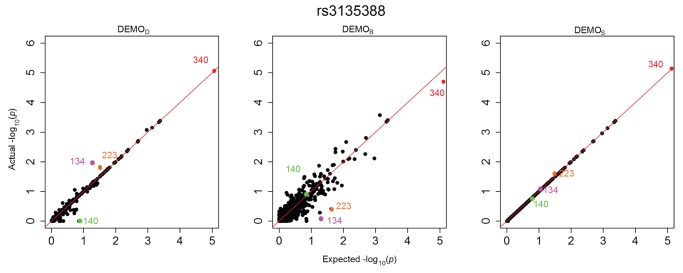
Changes in 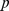-values for associations between clinical conditions and SNP rs3135388 presented as a QQ-plot for left) 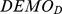, middle) 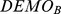, and right) 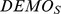. Descriptions of the annotated conditions in the plots are provided in Table 5.

**Figure 5:**
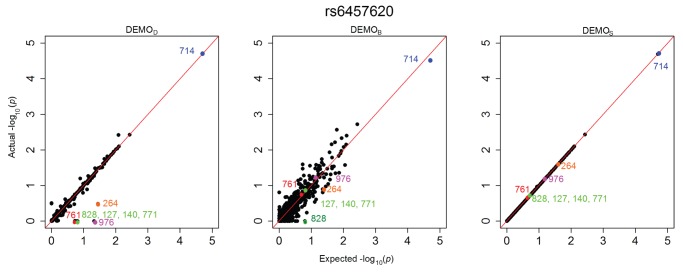
Changes in 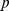-values for associations between clinical conditions and SNP rs6457620 presented as a QQ-plot for left) 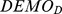, middle) 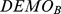, and right) 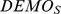. Descriptions of the annotated conditions in the plots are provided in Table 5.

**Figure 6:**
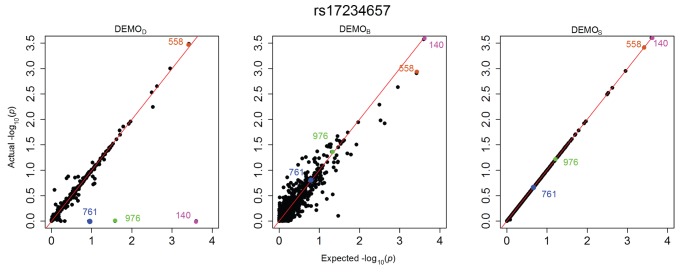
Changes in 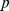-values for associations between clinical conditions and SNP rs17234657 presented as a QQ-plot for left) 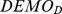, middle) 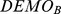, and right) 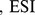. Descriptions of the annotated conditions in the plots are provided in Table 5.

Figure 1, Figure 2, Figure 3, Figure 4, Figure 5, and Figure 6 illustrate how 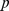-values change for the phenotype associations across all SNPs in the form of QQ-plots. A perfect similarity would be represented by the line 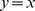, such that points along this line indicate the value in the original and anonymized analysis are equivalent. By contrast, points that deviate from this line indicate a change in the p value, such that the distance to the line indicates the magnitude of change. It can be seen that the 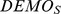 results lie consistently along the basis line, indicating that the values calculated in the original and anonymized PheWAS were approximately equivalent. For 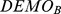 and 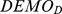, however, there are more differences in the 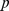-values between those derived from the original and anonymized datasets. Below, we highlight some specific changes in each PheWAS study conducted.

**Table 5:** Descriptions for the condition codes presented in the QQ-plots.

Code	Condition
127	Other intestinal helminthiases
134	Other infestation
140	Malignant neoplasm of lip
223	Benign neoplasm of kidney and other urinary organs
242	Thyrotoxicosis with or without goiter
264	Vitamin A deficiency
307.1	Eating disorders
392	Rheumatic chorea
411	Ischemic heart disease
458	Hypotension
558	Other and unspecified noninfectious gastroenteritis and colitis
676	Other disorders of the breast associated with childbirth and disorders of lactation
714	Rheumatoid arthritis and other inflammatory polyarthropathies
738	Other acquired musculoskeletal deformity
743	Congenital anomalies of eye
761	Fetus or newborn affected by maternal complications of pregnancy
762	Fetus or newborn affected by complications of placenta, cord, and membranes
764	Slow fetal growth and fetal malnutrition
771	Infections specific to the perinatal period
777	Perinatal disorders of digestive system
778	Conditions involving the integument and temperature regulation of fetus and newborn
779	Other and ill-defined conditions originating in the perinatal period
828	Multiple fractures involving both lower limbs, lower with upper limb, and lower limb(s) with rib(s) and sternum
976	Poisoning by agents primarily affecting skin and mucous membrane, ophthalmological, otorhinolaryngological, and dental drugs

**Figure 7:**
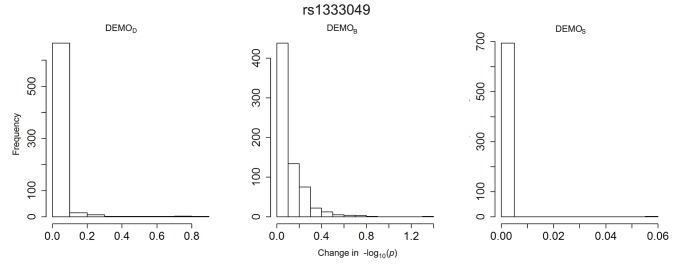
Distribution of 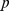-value changes for associations between clinical conditions and SNP rs1333049 for left) 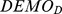, middle) 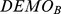, and right) DEMOS.

**Figure 8:**
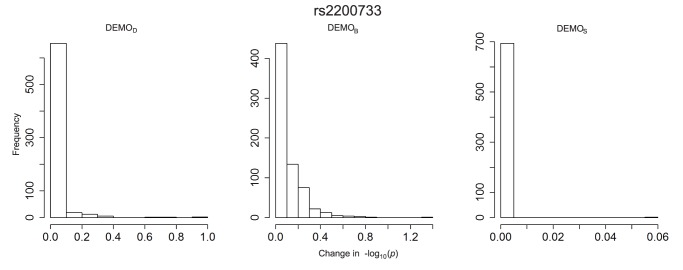
Distribution of 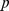-value changes for associations between clinical conditions and SNP rs1333049 for left) 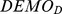, middle) 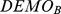, and right) DEMOS.

**Figure 9:**
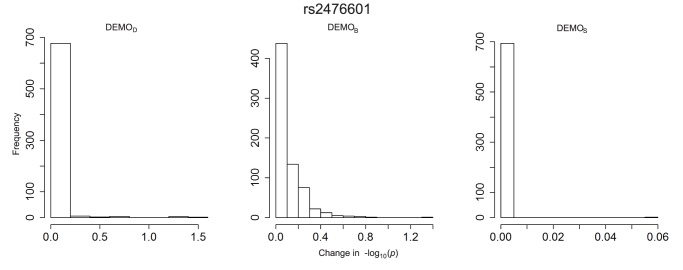
Distribution of 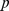-value changes for associations between clinical conditions and SNP rs2476601 for left) 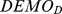, middle) 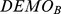, and right) 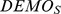.

**Figure 10:**
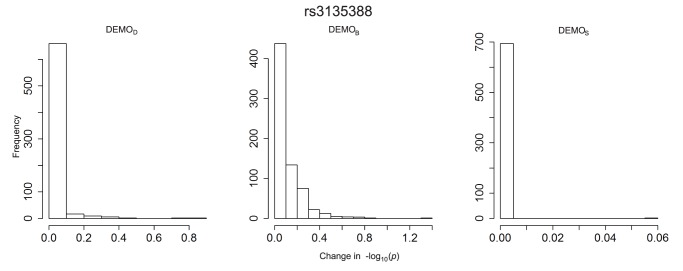
Distribution of 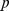-value changes for associations between clinical conditions and SNP rs3135388 for left) 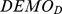, middle) 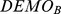, and right) 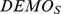.

**Figure 11:**
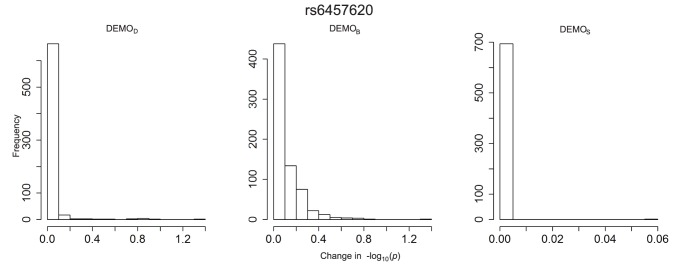
Distribution of 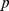-value changes for associations between clinical conditions and SNP rs6457620 for left) 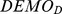, middle) 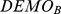, and right) 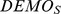.

**Figure 12:**
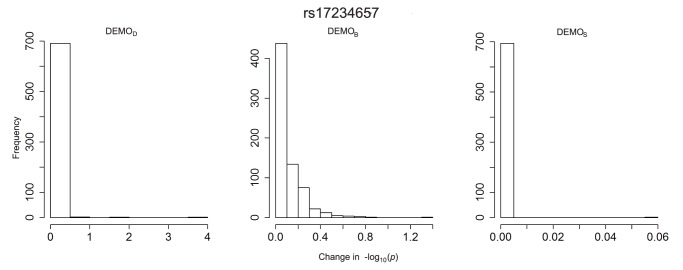
Distribution of 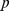-value changes for associations between clinical conditions and SNP rs17234657 for left) 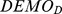, middle) 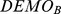, and right) 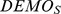.

The plots in Figure 7, Figure 8, Figure 9, Figure 10, Figure 11, and Figure 12 depict the change in 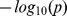-values. It can be seen that 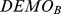 resulted in value changes of various frequencies, but that the 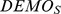 remained consistent across the association studies, with nearly 700 conditions (i.e., ICD-9 groupings) retaining their original 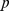-value. We can also see that 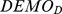, while having fewer and smaller changes, those changes were significant enough to alter the effects of the significant conditions.

#### rs1333049

Figure 1 indicates there were at least three associations expected between approximately 0.5 and 1. Yet, in the anonymized dataset, the 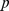-values for these associations were all close to 0, indicating a high likelihood of association. An example of a condition in this affected region is 440.00 *atherosclerosis*. There were also lost associations, such as condition 486 pneumonia, unspecified organism which, in the original PheWAS, was considered a significant association, but 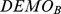 has a changed *p*-value such that the condition is now non-significant.

#### *rs*2200733

Figure 3 indicates there was an association which was anticipated to have a 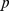-value of approximately 1.6. Yet, in the anonymized dataset, the 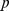-value for this association was close to 4, indicating a high likelihood of association.

#### rs2476601

Figure 2 indicates there was an association which was anticipated to have a 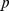-value of approximately 0.5. Yet, in the anonymized dataset, the 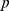-value for condition 762 was close to 2, indicating a high likelihood of association.

#### rs3135388

Figure 4 indicates that condition 134 would originally have a lower likelihood of association. However, the anonymization, its 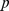-value has sufficiently changed for it to be considered significant. Similarly, in 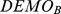, condition 223 originally had a value of approximately 1.6. In the anonymization, however, this value decreased to approximately 0.3, making it far less likely to be labeled significant.

#### rs6457620

Figure 5 indicates an expected value of at least 0.75 for a number of conditions, including 761, 976, 828, and 127, that, when anonymized, appear to be at or near 0.

#### rs17234657

Figure 6 indicates an expected value of at least 1.5 for two conditions, 976 (1.5) and 140 (3.6), when anonymized, appear to be at or near 0, completely removing them from significance.

### Summary of Findings

In terms of general information retention, 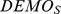 always outperformed 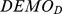 and 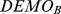. This result suggests that the larger the initial set from which the subset is drawn, the more likely it is that deidentification can retain associations that are being sought. In terms of PheWAS, 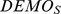 exactly matched the evaluation performed on DEMO the non-anonymized Demonstration cohort. We have also shown that, when compared to the total amount of information in the original data (i.e., the SD), 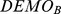 outperforms 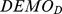, but that when anonymized data is compared to its non-anonymized data, the opposite is true in some of our measures. Additionally, 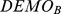 has a much greater number of lost and false associations than 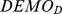 (or 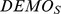).

**Figure 13:**
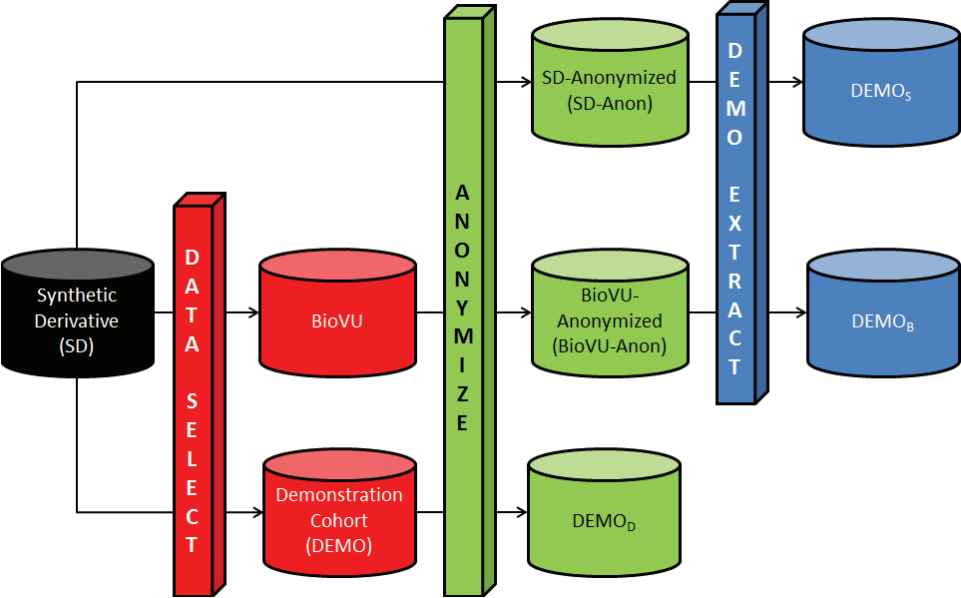
A fictional example of patient-specific records of diagnosis codes in the a) original resource and b) corresponding 2-anonymized result. The braces (“{ ldots }”) demarcate the set of diagnoses received in a visit to a healthcare provider, while the brackets (“
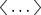
”) denote codes that have been generalized in accordance with the anonymization discussed herein.

The relationship between 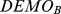 and 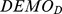 is not quite as paradoxical as it may appear. In BioVU, when generalizing codes, some records have repeated incidents of related, low-frequency conditions. For example, consider record 49532 in Figure 13a. Notice that in one visit, both codes 401.00– *malignant hypertension* - and 401.01– *benign hypertension* - are present. However, in Figure 13b, the codes have been replaced with 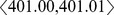 (read as: “400.00 and/or 400.01”). Considering just these codes, the DC in the original dataset equals two. However, once these codes are transformed into 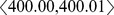, the result is a single generalized code, which halves the number of diagnoses in the anonymized dataset, yielding a DC of one. While the expectation was that BioVU would yield better results due to its significant increase in size, DEMOs population was selected to satisfy several specific phenotypes. As a result, records in DEMO were much more similar than records within BioVU. Consequently, less generalization was necessary to obtain 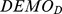 than 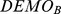.

This does not indicate that the anonymization strategy is ineffective. Instead, we have shown that it is important for the data holder to anticipate how the post-anonymization data will be used. If the data is intended to assist in hypothesis validation for a very specific cohort, then use of 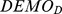 may be sufficient. If, however, the data is intended to support hypothesis generation, then the use of 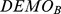 may be preferable. Regardless of the end use, however, 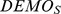 provides the most benefit to either task. As an additional merit to the use of 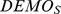, any further cohort that is drawn from SD-Anon is subject to the same protection, which means that if a user is grouped into two different cohorts, the exact same information will be revealed about this user to both groups. Separate anonymizations for data selections may not hold this property. Further research is necessary to determine what privacy claims, if any, may hold over repeated anonymizations of separate cohorts.

## Discussion

The anonymization method proposed in this paper is a significant improvement over prior approaches. It enables healthcare institutions to account for adversaries of varying strengths. Moreover, our analysis illustrates that when an adversary is aware that a patient was a member of the hospitals general population (as opposed to as a specific cohort), the utility of the anonymized cohort is virtually equivalent to the pre-anonymized results. These results suggest that when reasonable adversarial models are applied in the context of large medical facilities, phenome-wide annotation of clinical populations could be anonymized, allowing public sharing of such data, without sacrificing research findings. Adoption of such a principled approach could enable much greater utility of extant research data sets such as currently stored within dbGaP.

This finding indicates that rather than selecting the smallest possible subset of data that may need to be released, there is significant value in anonymizing the entire body of data at an institution. Release of even subsets of these data provide far more data to subsequent researchers, while still maintaining a high standard of privacy for the patients reflected in these data. This implies that institutions may be able to publicly release large, dense datasets for various research purposes with provable privacy guarantees.

Our study does include limitations, which can serve as guidelines for future research. First, from a technical perspective, the clinical code generalization strategy employed by the anonymization algorithm does not guarantee minimizing the amount of information loss incurred by the anonymization. For example, our algorithm chooses only one potential generalization from among many options. We chose this method of generalization because it is known to be computationally intractable for such data to be produced in a manner that minimizes information loss [46], [47]. Nonetheless, we suspect that additional heuristics may be devised which can provide improvements or alternatives to our results.

Second, from an implementation perspective, it is important to note that certain healthcare institutions may be more likely to be attacked than others. As a consequence, we recommend that a healthcare institution assess the anticipated capabilities of their data recipients before adopting an anonymization strategy such as the one presented in this manuscript. For instance, healthcare institutions may choose a weaker adversarial model if they anticipate that the data recipient is a credentialed scientific investigator as opposed to an unknown individual in the general public [33]. Similarly, healthcare institutions manage vastly different volumes of data. While we have shown here that the utility and privacy impact on data of this magnitude are beneficial, further work is needed to determine what volume of data is necessary to obtain similar findings.

## Methods

### Study Overview

**Figure 14:**
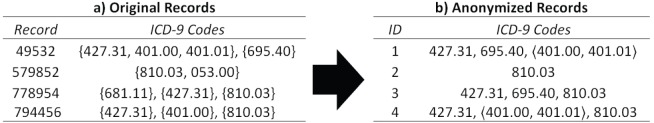
Datasets used for comparison of anonymization strategies. The DATA SELECT process is an extraction of some records of the SD into a smaller, specific dataset, such as BioVU or a demonstration cohort. The ANONYMIZE process is the anonymization algorithm described in this manuscript. The DEMO EXTRACT process selects the remaining records associated with the Demonstration cohort from a larger, anonymized dataset. The resultant datasets are as follows: anonymized version of the Synthetic Derivative (SD-Anon); anonymized version of BioVU (BioVU-Anon); SD-Anon, from which the demonstration group is extracted (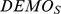); BioVU-Anon, from which the demonstration group is extracted (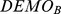); and the anonymized version of the demonstration cohort (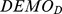). 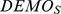, 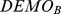, and 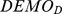 each represent different anonymizations of the Demonstration group.

A summary of the datasets analyzed in this study are reported in Table 1, while their relationships are visually depicted in Figure 14.

The first dataset corresponds to a HIPAA de-identified (see Methods) version of all VUMC patient records, called the Synthetic Derivative (SD) [48], which contains 1,366,786 records. The second dataset corresponds to a subset of this resource for which the VUMC collected de-identified DNA samples, called BioVU (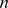 = 104,904). The third dataset, referred to as DEMO (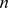 = 5,944), is a subset of BioVU records that were previously analyzed to demonstrate the feasibility of phenome-wide association studies (PheWAS), using specific genotypes, via information in existing EMRs [43]. In this dataset, each patient record is divided into a series of visits made to VUMC-affiliated healthcare providers. Each visit is characterized by the clinical activities that transpired, including diagnoses made, medications prescribed, and laboratory test results. An example of the structure of such records is depicted in Figure 13a. For this study, we anonymize the ICD-9 billing codes in the records, but we remark that our method is sufficiently general to apply to any standardized vocabulary of clinical events.

To model how cohorts are disseminated for validation and reuse, we developed a novel anonymization strategy that enables a subset of the SD to be shared for research purposes. In short, this strategy yields a patient record composed of diagnoses across all their visits. The information is anonymized, such that for any set of disclosed ICD-9 codes obtained at any one visit, there are at least *k* records in the anonymized resource with this combination of codes across all visits. For illustration, Figure 13b depicts a fictional example of anonymized records, with *k* set to 2. In our evaluation, we set *k* to 5, which is a level of protection commonly applied in practice [41].

There are several ways in which data can be anonymized to account for the knowledge of the recipient. Figure 14 depicts the various strategies. We begin with all data contained within the SD, from which we select two subsets. The first subset is BioVU and the second is DEMO. Each of the three datasets is then anonymized to create SD-Anon, BioVU-Anon, and 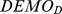, respectively. To examine the effect that each of the anonymizations have on subsequent analysis, we then extract the records which are in DEMO from SD-Anon and BioVU-Anon, creating 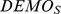 and 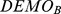, respectively. Note, 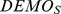, 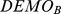, and 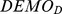 each contain the same records, but the specific clinical codes within those records are different due to the anonymization process.

### Privacy Models and Methods

There are a variety of computational models that have been proposed for protecting biomedical data. Most recently, randomization strategies, notably those based on differential privacy [49], [50], have been suggested. These approaches perturb records through a controlled, but random, process (e.g., addition of codes not originally diagnosed). Such a framework provides strong proofs of privacy, but may be insufficient to support new studies at varying levels of granularity. Moreover, if care is not taken in its design, this strategy could lead to strange data representations (e.g., juvenile patients diagnosed with Alzheimers disease), and in the co-occurrence with a chance rare genetic event (e.g., a rare functional mutation in an exon), could lead to an erroneous association. Thus, we focused on data protection models that remain true to the underlying data. To do so, we adopted a variation of the *k*-anonymization principle [51], which states that any combination of potential identifiers in the resultant dataset must match at least *k* records. This principle has been applied to various types of patient-level data, such as demographics [41], as well as clinical codes [40]. To achieve privacy in our setting, we enforced a constraint which states that, for each visit of a patient, there are at least *k* patients who have the same set of diagnosis codes from some visit in the resulting dataset. This model allows us to represent an adversary with a moderate, but manageable, level of knowledge regarding patient information released by the institution.

### Health Data De-identification According to Federal Regulation and Residual Risks

Our goal is to enable biomedical analysis with patient-level records while thwarting re-identification attempts. Returning to Figure 13, the SD is de-identified according to the Privacy Rule of the Health Information Portability and Accountability Act of 1996 (HIPAA). This was accomplished by removing eighteen specific features of the data, including direct identifiers (e.g., patient names and residential address), quasi-identifiers (e.g., dates of birth, death, and healthcare provider visits), and specific identification numbers or codes (e.g., medical device identification numbers). Despite the removal of such information, many records may be uniquely distinguishable based on the combination of their diagnosis codes. [31] For instance, imagine that an attacker knows a patient, say “Alice” (49532), was assigned billing codes 427.31 *atrial fibrillation* and 401.00 *hypertension* in a hospital visit. Then, according to the depiction to the left of Figure 13, Alice will be uniquely identified in the original dataset. This means that the attacker learns Alice was additionally diagnosed with code 695.40 *systemic lupus* (as well as any other codes or DNA sequences in the released dataset). However, in the anonymized version of the table to right of Figure 13, an attacker would be unable to determine whether this patient is Record 1 or Record 4.

### Clinical Concept Anonymization Process

To satisfy anonymization requirements, we invoke a system of code generalization. The generalization replaces a specific ICD-9 code with a group of codes which are semantically similar. For example, to successfully anonymize a dataset, we may need to generalize the code 810.01 *closed fracture of sternal end of clavicle* to the code “810.00 and/or 810.01” *closed fracture of clavicle, sternal and/or unspecified*. However, this generalization introduces the need for guidelines on what codes may acceptably be generalized together, which are called utility constraints. For instance, generalizing 810.01 to “940 and/or 810.01” *burns* or *closed sternal fracture of the clavicle* would have introduced an entirely new condition “burns” instead of simply obscuring the specific region of the clavicle which had been broken. To prevent such occurrences, we use a hierarchy that defines what generalizations are allowed. For this work, we use the hierarchy described in [43], which is a complete mapping of all ICD-9 codes developed for clinical phenotype-genotype association studies.

For the anonymization, our approach generalizes ICD-9 codes with frequency (i.e., the number of records that contain the code in one or more visits) below a threshold *k* within the dataset together. First, we place each code in a bin corresponding to its frequency. For instance, all codes assigned to only one patient are stored in the first bin. For each bin with value less than k, the process generalizes the codes within that bin as permitted by the utility constraints. Next, the support is calculated for the new, generalized code, which is moved into the appropriate bin. On each subsequent iteration, we group adjacent bins together (i.e., bins one and two are grouped together, bins three and four are grouped together) until all bins representing frequency less than *k* have been grouped together. Note that by merging adjacent bins (such as one and two), the result does not necessarily get moved into bin three. Instead, the frequency is recalculated for all patients who would have the new, generalized code. After this point, any codes remaining with frequency less than *k* are suppressed.

At this point, the dataset satisfies the *k*-anonymization requirement and can be shared. However, assuming that the institution holding the data does not wish to release the entire anonymized dataset, this is the point at which subsets may be drawn from the data. For example, suppose that external researchers were interested in patients who had ischemic heart disease. Records of specific interest could be extracted from the anonymized data and then released to these researchers.

### Computation of Diagnosis Count and Code Count

**Figure 15:**
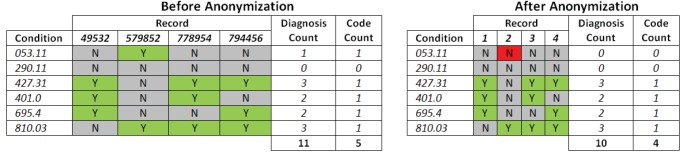
An illustration of the computation for Code Count and Diagnosis Count. The table to the left illustrates if a record contained a condition (green “Y”) or did not (grey “N”) in the original dataset before anonymization. The table to the right illustrates if a record lost a diagnosis (red “N”). Notice that the second record lost one diagnosis, which resulted in both the Code Count and Diagnosis Count to be lowered by a score of 1.

In Figure 15, we show an example computation of Diagnosis Count (DC) and Code Count (CC). In the left part of the figure, we show a non-anonymized example data set containing six conditions, 053.11, 290.11, 427.31, 401.0, 695.4, and 810.03, and four records - A, B, C, and D. We represent the datum that Record B was diagnosed with condition 053.11 in some visit as a “Y” (also shown as a green cell highlight). The absence of this diagnosis is represented as an “N” (also shown as a grey cell highlight), as shown in the cell represented by condition 053.11 and Record A.

As shown, Diagnosis Count is simply the number of times that a particular diagnosis is assigned across all patients in the set. Since Record B is the only one that contains the diagnosis 053.11, its Diagnosis Count is 1. Alternatively, Code Count is a count of the number of codes that have positive diagnoses in that data set. As shown in Figure 15, code 290.11 has no records with that diagnosis. As such, its Code Count is 0. Since each other code has at least one record with a positive diagnosis, each other count is 1, giving the data set a Code Count of 5.

On the right side of Figure 15, we show a possible change in our measures following anonymization. In this instance, the anonymization has suppressed Record 2’s diagnosis of 053.11 (now represented as “N” in a red cell highlight). Because this decreased the number of diagnoses in the data set, the Diagnosis Count decreased by 1. However, since this was also the only diagnosis of condition 053.11, that code is no longer represented in the data; thus, the Code Count also decreased by 1.
