# Correction: Enabling Genomic-Phenomic Association Discovery without Sacrificing Anonymity

**DOI:** 10.1371/annotation/db45627a-50ec-4c52-8b5f-95dec75b1c80

**Published:** 2013-06-18

**Authors:** Raymond D. Heatherly, Grigorios Loukides, Joshua C. Denny, Jonathan L. Haines, Dan M. Roden, Bradley A. Malin

The order of figures 13 and 14 is switched. 

